# Formulae for generating standard and individual human cone spectral sensitivities

**DOI:** 10.1002/col.22879

**Published:** 2023-07-19

**Authors:** Andrew Stockman, Andrew T. Rider

**Affiliations:** ^1^ Institute of Ophthalmology University College London London UK; ^2^ State Key Laboratory of Modern Optical Instrumentation Zhejiang University Hangzhou China

**Keywords:** CIE standards, color matching functions, cone fundamentals, cone spectral sensitivity functions, individual differences

## Abstract

Normal color perception is complicated. But at its initial stage it is relatively simple, since at photopic levels it depends on the activations of just three photoreceptor types: the long‐ (L‐), middle‐ (M‐) and short‐ (S‐) wavelength‐sensitive cones. Knowledge of how each type responds to different wavelengths—the three cone spectral sensitivities—can be used to model human color vision and in practical applications to specify color and predict color matches. The CIE has sanctioned the cone spectral sensitivity estimates of Stockman and Sharpe (Stockman and Sharpe, 2000, Vision Res) and their associated measures of luminous efficiency as “physiologically‐relevant” standards for color vision (CIE, 2006; 2015). These LMS cone spectral sensitivities are specified at 5‐ and 1‐nm steps for mean “standard” observers with normal cone photopigments and average ocular transparencies, both of which can vary in the population. Here, we provide formulae for the three cone spectral sensitivities as well as for macular and lens pigment density spectra, all as continuous functions of wavelength from 360 to 850 nm. These functions reproduce the tabulated discrete CIE LMS cone spectral sensitivities for 2‐deg and 10‐deg with little error in both linear and logarithmic units. Furthermore, these formulae allow the easy computation of non‐standard cone spectral sensitivities (and other color matching functions) with individual differences in macular, lens and photopigment optical densities, and with spectrally shifted hybrid or polymorphic L‐ and M‐cone photopigments appropriate for either normal or red‐green color vision deficient observers.

## Graphical abstract

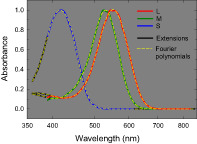

## 1 INTRODUCTION

Color perception depends on complex spatial and temporal interactions occurring within serial and parallel neural processing networks, but the initial stage of processing is relatively simple since it depends only on the relative activations of three types of cone photoceptor: the long‐ (L‐), middle‐ (M‐) and short‐ (S‐) wavelength‐sensitive cones. The three cone types all contain the same chromophore, 11‐*cis* retinal, but bound to different photopigment opsins. Upon photon absorption, 11‐*cis* retinal changes isoform to all‐*trans* retinal and thereby initiates the phototransduction cascade. The probability of photon absorption as a function of wavelength and thus the cone spectral sensitivity is modified by the photopigment opsin.

Measured with respect to the sensitivity to lights of different wavelengths entering the eye at the cornea, the “corneal cone spectral sensitivities” depend on the cone spectral sensitivities at the retina and the filtering by the lens and macular pigments through which the light must pass before reaching the photopigment; for review, see Reference [1]. All these factors are subject to individual variability, so that different observers viewing the same scene may make different color matches and perceive different colors. Here, we provide a simple means of computing corneal cone spectral sensitivities that can incorporate individual differences and thus predict color matches for non‐standard observers.

The Commission Internationale de l'Éclairage (CIE) sanctioned the cone spectral‐sensitivity estimates of Stockman and Sharpe^2^ and their associated measures of luminous efficiency^3^, ^4^ as “physiologically‐relevant” standards for color vision.^5^, ^6^ The standards are tabulated for 2‐deg and 10‐deg visual fields at 5‐nm steps in CIE publications^5^, ^6^ and at 0.1, 1, and 5‐nm steps on www.cvrl.org (at up to eight decimal places to reduce rounding errors in calculations). They are arguably the most secure estimates of mean human cone spectral sensitivities available for modeling human color vision since they are based on observers of known genotype.^7^ They are called the standard cone fundamentals for reasons explained later. They follow a long history of cone spectral sensitivity estimates, the first plausible estimates of which were obtained in the 19th century by König and Dieterici.^8^ Notable estimates since then include those by Bouma,^9^ Judd,^10^, ^11^ Wyszecki and Stiles,^12^ Vos and Walraven,^13^ Smith and Pokorny,^14^ Vos,^15^ Estévez,^16^ Vos, Estévez and Walraven,^17^ and Stockman, MacLeod and Johnson.^18^ These studies generally aimed to exclude anomalous observers; that is, observers known now to have L/M hybrid opsin genes the spectral sensitivities of which are shifted and lie between the standard L‐ and M‐cone fundamentals. Stockman and Sharpe^2^ were able to exclude anomalous observers on molecular genetic grounds.

Here, we develop mathematical descriptions of the standard cone fundamentals that are continuous functions of wavelength and can replace the discrete, tabulated functions with very low residual error (see Figure 3, below). These continuous functions relate to the underlying photopigment absorbance spectra and thus can incorporate pre‐receptoral filtering, so enabling fundamentals with normal or shifted *λ*_max_ values (the wavelength at which cone sensitivity is maximal) and different macular, lens, and photopigment optical densities to be easily generated.

We start by generating continuous representations that define the cone spectral sensitivities for the mean standard normal observer for 2‐ and 10‐deg vision. Our strategy is to fit functions to the L‐, M‐ and S‐cone photopigment absorbance spectra and the standard lens and macular optical density spectra and from them to calculate the corneal cone fundamentals (the corneal cone spectral sensitivities).

We will consider the representation of the cone fundamentals in other color spaces in a separate section below.

### 1.1 Glossary

**2‐deg:** Small‐field colour matches made with centrally viewed, circular fields subtending 2‐deg diameter of visual angle.

**10‐deg:** Large‐field colour matches made with centrally viewed, circular fields subtending 10‐deg diameter of visual angle.

**Chromaticity coordinates:** *l, m,* which in terms of the tristimulus values are *L*/(*L* + *M* + *S*) and *M*/(*L* + *M* + *S*), respectively (*r*, *g* for **RGB** space, or *x*, *y* for **XYZ** space).

**CIE:** Commission Internationale de l'Éclairage or International Commission on Illumination. An organization that sets international standards for colour and lighting.

**Colour match:** A subjective match between two lights, typically side‐by‐side semi‐circular fields, of different spectral power distributions (which are consequently metamers).

**Colour matching functions:** $\bar{r}\left(\lambda \right)$, $\bar{g}\left(\lambda \right)$, $\bar{b}\left(\lambda \right)$. Tristimulus values of the equal‐energy, monochromatic spectrum locus.

**Colorimetry:** The measurement and specification of colour.

**Cone fundamentals:** Cone spectral sensitivities: $\bar{l}\left(\lambda \right)$, $\bar{m}\left(\lambda \right)$ and $\bar{s}\left(\lambda \right)$in colorimetric notation. These are the colour matching functions for three imaginary primaries, **L**, **M** and **S**, that uniquely stimulate each of the cones.

**Imaginary light:** Theoretical light that has negative energy in some region of the visible spectrum. Examples include the imaginary cone primaries **L**, **M** and **S** with spectral power distributions $L\left(\lambda \right)$, $M\left(\lambda \right)$ and $S\left(\lambda \right)$, respectively.

**Metamers:** Two lights that match, but which are physically different. An example is a match between a yellow light and a mixture of red and green lights.

**Photopigment absorbance spectra:** Relative probability of photon absorption as a function of wavelength for the different cone photopigment opsins. These are hypothetical functions that correspond to the absorbance probability of an infinitely dilute solution of photopigment. Also known as extinction spectra.

**Photopigment optical density:** Attenuation of light per unit length of photopigment as a function of wavelength. It depends on the photopigment absorbance spectra, photopigment density, and the axial length of the cone outer segment.

**Photometry:** The measurement and specification of the luminous efficiency of lights intended to be independent of colour.

**Photopic luminosity function:** Photometric measure of luminance efficiency as a function of wavelength under photopic (*i.e*., rod‐free) conditions: $V\left(\lambda \right)$or $\bar{y}\left(\lambda \right)$.

**Primary lights: R, G, B**. The three independent primaries (real or imaginary) to which the test light is matched (actually or hypothetically). They must be independent in the sense that no combination of two can match the third.

**Spectral power distribution:** $P\left(\lambda \right)$. The power of a light, **P**, as a function of wavelength.

**Standard observer:** The standard, mean observer is the hypothetical person whose colour matching behavior is represented by a particular set of mean CMFs.

**Trichromacy:** The ability of normal observers to match test lights with a mixture of three independent primary lights.

**Tristimulus values:** *R, G, B,* the amounts of the three primaries required to match a given stimulus.

**Univariance:** The output of a photoreceptor varies *unidimensionally* according only to the rate of photon absorption.

## 2 FORMULAE FOR STANDARD OBSERVERS

### 2.1 Normal cone photopigment absorbance spectra

The templates used for the photopigment absorbance spectra are effectively 8th order Fourier polynomials (sometimes known as truncated Fourier series) of the form:

(1) $$F\left(\theta_{P}\right)=a_{0}+\sum_{k=1}^{8}\left[a_{k}\cos\left(k\theta_{P}\right)+b_{k}\sin\left(k\theta_{P}\right)\right]+s.$$

It is important to note that this function is purely descriptive and, as shown below, provides a very good fit to the data, but there is no theoretical significance to the function or any of the parameters (the value *s* is a renormalization factor added after the polynomial fit so that the linear absorbance spectra peak exactly at 1 at *λ*_max_ [to the nearest 0.1 nm]). The variable *θ*_*P*_ varies from 0 to *π* over the fitted wavelength region and corresponds to one half period of the fundamental of the Fourier polynomial. We used a log wavelength scale from 2.556 and 2.929 log_10_ nm (360–850 nm) and only half the period of the fundamental because the absorbance spectra are not equal at their long‐ and short‐wavelength ends and using the full 2π period of *θ*_*P*_ would force them to be equal. For *θ*_*P*_ to vary from 0 to *π* over the log_10_(360) to log_10_(850) range:

(2) $$\theta_{P}=\pi \frac{\log_{10}\left(\lambda /360\right)}{\log_{10}\left(850/360\right)}.$$

A log wavelength scale was used because the shapes of the main peak of the photopigment absorbance spectra (known as the “*α*‐band”) are approximately invariant across different λ_max_ when plotted against such a scale.^19^, ^20^, ^21^ Thus, this scale can straightforwardly be used to shift the cone absorbance spectra to account for small individual differences in λ_max_ without changing the shape of the spectrum for different *λ*_max_ (however, as we show below, the spectra for the different cone types do vary slightly in shape even on a logarithmic wavelength scale, so that the formulae for the L‐ M‐ and S‐cone spectra have been separately determined).

We start with the absorbance spectra adopted by the CIE from Stockman and Sharpe.^2^ These spectra range from 390 to 830 nm for the L‐ and M‐cone spectra and from 390 to 615 nm for the S‐cone spectrum. The range corresponds to the range of wavelengths used by Stiles and Burch in their 10‐deg color matching experiments. The upper limit of the S‐cone spectrum corresponds to the longest wavelength measured in S‐cone monochromats.^22^ Because we also want to be able to spectrally shift the cone fundamentals to account for individual differences in λ_max_, we have extended the absorbance spectra by extrapolating them all to 360 nm at short wavelengths and to 850 nm at long wavelengths. The short wavelength region from 360 to 400 nm is the upper end of the ultra‐violet spectrum (UVA), which is usually taken to extend from 315 to 400 nm^23^ and the long wavelength region from 780 to 850 nm falls in the near infrared region (NIR or IR‐A).

The long‐wavelength extrapolations were achieved by aligning Lamb's photopigment template^21^ with the existing data and extending the data from 830 to 850 nm for the L‐ and M‐cone spectra and from 615 to 850 nm for the S‐cone spectrum. Although the extensions are very plausible,^24^ it is not crucial that they are exactly correct, since they lie at the spectral extremes for each photopigment where they have little effect on typical color matching predictions.

The short‐wavelength extrapolations are more problematic for several reasons. First, because there is a lack of normal color matching data in that region. Second, because the lens and macular densities below 390 nm are uncertain. Third, because any color matching measurements in that region are affected by the fluorescence of the lens and cornea (see for review^25^), as a result of which the usual laws that underlie color matching^26^ may not apply. Another concern is that the CIE L‐ and M‐cone photopigment absorbance spectra proposed by Stockman and Sharpe^27^ and adopted by the CIE may be incorrect below 400 nm, since between 400 and 390 nm the absorbance spectra fall, whereas other estimates of photopigment absorbance spectra suggest that they should rise slightly between 400 and 390 nm—consistent with the shallow, secondary “*β*‐band” peak in photopigment spectra at shorter wavelengths.^24^, ^28^ Given the uncertainties about both lens densities and color matches below 400 nm, we have taken the somewhat drastic step of adjusting the CIE spectra from 390 to 400 nm to be consistent with spectra derived from photopigment measurements. To achieve this, we calculated a shallow *β*‐band spectrum for the L‐cone photopigment below 400 nm using the photopigment template formula proposed by Govardovskii et al.,^24^ and aligned it with the original CIE L‐cone photopigment absorbance spectrum at 400 nm. The M‐cone spectrum was then approximated below 400 nm by appropriately spectrally shifting the modified L‐cone spectrum. The S‐cone spectrum was extended below 390 nm by shifting the new L‐ and M‐cone spectra to align with the S‐cone spectrum at 390 nm and then averaging the aligned templates. Shifts were carried out along a logarithmic wavelength axis. In terms of the CIE standards, we have therefore slightly changed the color matching data between 390 and 400 nm. These changes have relatively little effect on most color matching predictions. Color in the UVA part of the spectrum is considered further in the Discussion.

**FIGURE 1:**
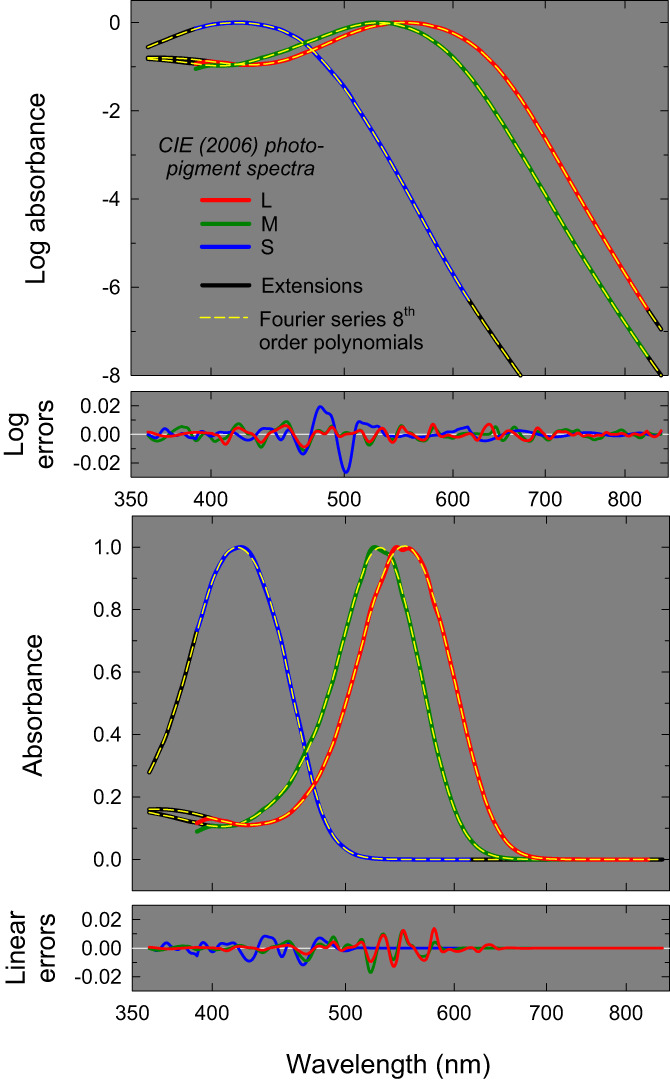
Logarithmic (top panel) and linear (third panel) L‐, M‐, and S‐cone photopigment absorbance spectra on a logarithmic wavelength scale (red, green, and blue solid lines, respectively) defined in the CIE 2006 standard from Stockman and Sharpe.^2^ The spectra have been extended to 360 and 850 nm (solid black lines) and the L‐ and M‐cone spectra have been modified slightly between 390 and 400 nm to accommodate a short‐wavelength extension consistent with other photopigment measurements. The 8th order Fourier polynomials that best fit the extended spectra are shown by the yellow dashed lines. See text for details. Errors in the fitted functions compared to the CIE 2006 standards are shown on logarithmic and linear scales in the second and fourth panels, respectively.

Figure 1 shows the discrete points of the tabulated CIE absorbance spectra connected by the red, green, and blue colored lines for the L‐, M‐ and S‐cone spectra, respectively. The logarithmic cone absorbance spectra are plotted in the upper panel and the linear absorbance spectra in the third panel. The spectra are normalized to have the same peak values. The absorbances are plotted as a function of wavelength shown on a logarithmic scale. The short‐ and long‐wavelength extensions are shown as the solid black lines. The dashed yellow lines show the fits obtained with Equations (1) and (2) (as outlined above, and evident in the linear representation, the short‐wavelength extensions for the L‐ and M‐cone spectra deviate from the CIE estimates below 400 nm).

We fitted Equations (1) and (2) to the logarithmic cone absorbance spectra from 360 to 850 nm, simultaneously minimizing both the logarithmic and linear errors. As the absorbances vary with wavelength over greater than 6 decades, minimizing the errors in just the linear absorbances led to substantial errors in the prediction of the smaller logarithmic absorbances and minimizing just the logarithmic errors led to substantial errors in the prediction of larger linear absorbances. To mitigate this problem, we simultaneously minimized both the logarithmic and the linear errors. All fits were made at 1‐nm steps using the standard non‐linear fitting Marquardt–Levenberg algorithm implemented in SigmaPlot (Systat Software, San Jose, CA) to minimize the sum of the squared differences between the data and model predictions. Separate fits were made to the L‐, M‐ and S‐cone fundamentals, the results of which are shown by the yellow dashed lines in top and third panels of Figure 1. We found that increasing the order of the Fourier polynomials beyond 8 did not significantly improve the fits in Figures 1 and 4, nor improve the reconstruction of the cone fundamentals and their transformation into other spaces such as the RGB and XYZ spaces shown in Figure 6, below. The polynomial coefficients for the three log cone absorbance spectra are given in Table 1. The continuous logarithmic cone absorbances as a function of wavelength, $\log_{10}\left({\bar{l}}_{A}\left(\lambda \right)\right)$, $\log_{10}\left({\bar{m}}_{A}\left(\lambda \right)\right)$, and $\log_{10}\left({\bar{s}}_{A}\left(\lambda \right)\right)$, are thus defined by substituting Equation (2) into Equation (1) with Fourier coefficients from the appropriate column in Table 1, and the corresponding linear absorbances, ${\bar{l}}_{A}\left(\lambda \right)$, ${\bar{m}}_{A}\left(\lambda \right)$, and ${\bar{s}}_{A}\left(\lambda \right)$, are found by taking antilogs.

**TABLE 1:** Fourier coefficients for the logarithmic cone absorbance spectra.

Fourier coefficients	L	M	S
*a*_0_	−42.926358	−210.656885	207.388095
*a*_1_	−2.039680	−0.145807	−6.306562
*b*_1_	75.971783	386.731976	−393.710048
*a*_2_	57.330821	305.471058	−315.665060
*b*_2_	6.573391	5.021838	19.291754
*a*_3_	8.111103	6.838622	19.641474
*b*_3_	−38.765649	−208.206234	214.221157
*a*_4_	−21.448345	−118.489020	121.858468
*b*_4_	−5.939747	−5.762587	−15.182074
*a*_5_	−3.389620	−3.797355	−8.677406
*b*_5_	9.588300	55.180346	−56.759638
*a*_6_	3.250756	19.972851	−20.631872
*b*_6_	1.441277	1.899046	3.693488
*a*_*7*_	0.396600	0.691341	1.048302
*b*_*7*_	−0.711392	−5.089181	5.365662
*a*_*8*_	−0.079354	−0.707069	0.789878
*b*_*8*_	−0.072980	−0.141993	−0.148036
*s*	−0.001655	0.000589	0.000236

Note that this procedure and the resulting formulae can be easily applied to other estimates of the cone spectral sensitivities, such as those by Smith and Pokorny,^14^ by first converting them to cone absorbance spectra; see appendix A of Reference [29].

The Fourier polynomials fit the cone absorbances extremely well on both logarithmic and linear scales. The logarithmic errors are shown in the second panel and the linear errors in the bottom panel. Most of the errors on both scales are less than ±0.01. There is a slight perturbation in the CIE S‐cone log absorbance spectrum between about 480 and 510 nm, which is smoothed by the fitted template. The adjusted *R* ^2^ values for the simultaneous fits are >99.99% for each of the L, M and S fits, and the standard errors of the L, M, and S fits are 0.003, 0.003 and 0.004, respectively. Thus, the errors resulting from the use of the proposed templates rather than the CIE tabulated functions will be small even for monochromatic lights.

The *λ*_max_ values of the photopigment spectra derived from Equations (1) and (2) using the values given in Table 1 are 551.9, 529.8 and 416.9, nm for L‐, M‐ and S‐cones, respectively. Note that the Fourier polynomial coefficients have little theoretical significance and can change substantially with small adjustments to the underlying spectrum being fitted. Their utility is in allowing continuous spectra to be computationally generated with negligible differences from the discrete, tabulated CIE functions (see Figure 3, below). They also provide continuous functions to allow arbitrary wavelength shifts associated with different values of *λ*_max_. Later, we consider the derivation of a common template to describe all three spectra.

### 2.2 Macular and lens optical density spectra

The templates used for the macular and lens pigment optical density spectra were also Fourier polynomials:

(3) $$F\left(\theta \right)=\left\{a_{0}+\sum_{k=1}^{n}\left[a_{k}\cos\left(k\theta \right)+b_{k}\sin\left(k\theta \right)\right]\right\}d,$$

where *n* = 11 for the macular spectrum and *n* = 9 for the lens spectrum. We used a linear wavelength scale to define both the macular and lens density spectra. The value *d* is a renormalization factor that scales the polynomials so that, consistent with the CIE standard, the macular spectrum is 0.350 at 460 nm and the lens spectrum is 1.7649 at 400 nm. Note that the densities are logarithmic—a density of 0.0 at any wavelength means it is perfectly transparent at that wavelength and a density of 0.350 at 460 nm means the macular pigment allows only 10^−0.350^ (44.7%) of light at this wavelength to pass through. A density of 1.7649 at 400 nm means the lens blocks all but 10^−1.7649^ (1.7%) of light at that wavelength.

**FIGURE 2:**
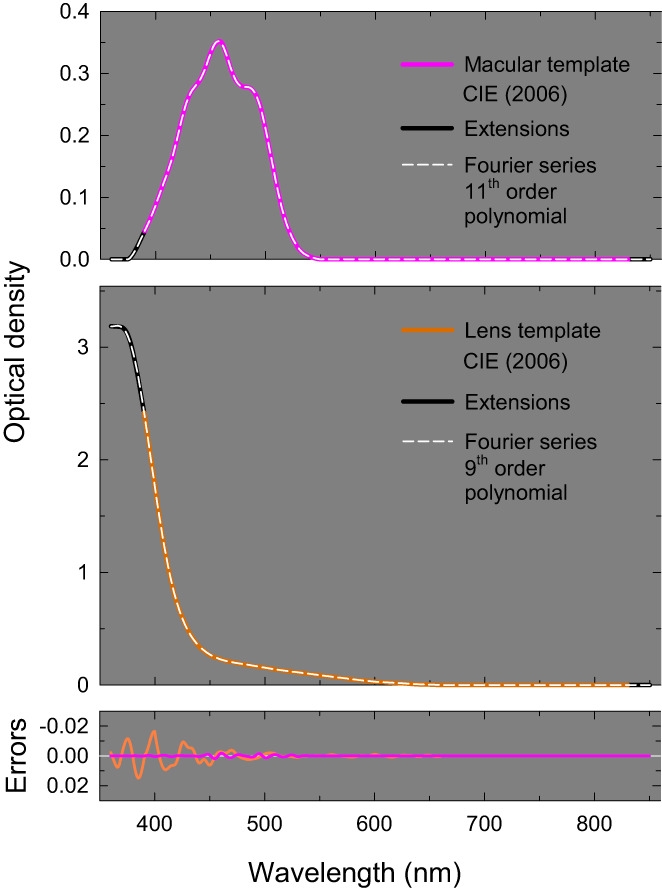
Standard macular optical density spectrum (upper panel, pink solid line) and lens pigment optical density spectrum (middle panel, orange solid line) extrapolated from the Stockman and Sharpe and CIE color matching standard with best‐fitting 11th order (macular) or 9th order (lens) Fourier polynomials (white dashed lines). See text for details. Errors in the fitted functions for macular (pink solid line) and lens (orange solid line) are shown in the bottom panel.

The macular pigment absorbs light mainly of short wavelengths (see Figure 2, top panel for 2‐deg visual fields; in both panels of Figure 2, wavelength is plotted on a linear scale). The pigmented macular area varies in shape from individual to individual and is irregular in density: greatest in the fovea and falling irregularly with retinal eccentricity to become largely absent by about 10 deg.^30^ The CIE standard macular density spectrum was originally proposed by Stockman and Sharpe^2^ based on measurements by Bone, Landrum and Cairns.^31^ The standard (mean) macular densities at 460 nm are assumed to be 0.350 and 0.095 for 2‐deg and 10‐deg vision, respectively.^2^

The wavelength range for the macular template fit was from 375 to 550 nm; outside this range the density is assumed to be zero. For *θ*_mac_ to vary from 0 to *π* as *λ* varies from 375 to 550 nm:

(4) $$\theta_{\mathrm{mac}}=\pi \frac{\lambda -375}{550-375}.$$

The standard CIE macular density template, like the L‐ and M‐cone absorbance spectra, is limited to between 390 and 830 nm. To extend this template to shorter wavelengths, we assumed that the density falls smoothly to zero between 390 and 375 nm and then remains at zero at shorter wavelengths. Wald^32^ made sparsely sampled spectral macular density measurements showing that at 365 nm the density was zero (see his figure 4). At wavelengths longer than 830 nm, we assumed that the template remains at zero. The short wavelength extrapolation is speculative, since most of the available data are limited to wavelengths of 400 nm and longer. In the top panel of Figure 2, the CIE macular template for 2‐deg vision is shown by the pink line and the extensions by the solid black lines.

We found that an 11th order Fourier polynomial captured macular optical density spectra well enough to avoid significant discrepancies from the original template. The fit is shown by the dashed white line in the upper panel. The errors, which are shown as the pink curve in the bottom panel, are very small. The adjusted *R* ^2^ value is >99.99% the standard error of the fit is 0.0007. The errors resulting from the use of this template rather than the original discrete tabulated functions are negligible. Higher order polynomials did not perform significantly better. The polynomial coefficients are given in Table 2. The macular pigment density as a function of wavelength, $\text{mac}\left(\lambda \right)$, is given by substituting Equation (4) into Equation (3) with the appropriate values from Table 2.

**TABLE 2:** Fourier coefficients for the macular and lens density spectra.

Fourier coefficients	Macular	Lens
*a*_0_	3712.203779	−313.950863
*a*_1_	374.181158	−70.321682
*b*_1_	−7007.698964	585.471973
*a*_2_	−5887.285752	471.539586
*b*_2_	−633.047523	117.353910
*a*_3_	−716.042904	127.016822
*b*_3_	4386.881125	−324.470054
*a*_4_	2882.109266	−188.163808
*b*_4_	638.134755	−104.551249
*a*_5_	468.498070	−68.307849
*b*_5_	−1653.756739	89.781537
*a*_6_	−817.124090	33.449826
*b*_6_	−286.403898	35.272364
*a*_7_	−144.799646	13.652409
*b*_7_	340.336483	−8.756817
*a*_8_	115.565280	−1.282577
*b*_8_	59.165083	−3.512653
*a*_9_	18.667820	−0.447784
*b*_9_	−30.234454	0.042829
*a*_10_	−5.468375	
*b*_10_	−4.133506	
*a*_11_	−0.504396	
*b*_11_	0.509417	
*d*	1.005005	1.009187

The lens contains a pigment that absorbs light mainly of short wavelengths (see Figure 2, middle panel). The standard CIE lens density spectrum,^5^ which is assumed to be 1.7649 at 400 nm, is shown by the orange solid line in the middle panel of Figure 2. It is a slightly adjusted version of the mean lens density spectrum of van Norren and Vos^33^ proposed originally by Stockman, Sharpe and Fach.^22^ Like the macular spectrum, it is defined from 390 to 830 nm. For consistency with the other templates, we have extended it down to 360 nm and up to 850 nm. Above 830 nm, we can reasonably assume that the density remains zero. Below 390 nm, however, the change in density with decreasing wavelength is highly uncertain, since the relatively few measurements of long‐wave UVA absorbance by the human lens that are available are inconsistent.^34^, ^35^, ^36^, ^37^

Cooper and Robson,^35^ for example, found that lens density in the UVA varied with age but was surprisingly high (4.5 log units at 360 nm in a 33‐year‐old lens). By contrast, Lerman and Borkman^36^ found that the density in the UVA did not vary with age between 25 and 82 years yet was implausibly low (on average about 0.7 log unit). Subsequently, Weale,^37^ who tried to resolve these discrepancies, found densities that were intermediate between these two extremes and confirmed that they did vary with age. In general, lens density in the UV increases substantially with age and peaks at around 360 nm and then falls with a window of reduced density centered on about 320 nm.^34^, ^35^, ^36^, ^37^

To extend the lens density template below 390 nm, we have used a slightly smoothed version of the lens density template proposed in another CIE report to facilitate computations of the absorption and transmission characteristics of the eye published in 2012.^38^ This template, which was based on measurements in rhesus monkey,^39^, ^40^ is broadly consistent with the human measurements of Weale.^37^ We have scaled the CIE 2012 lens density spectrum to align with the CIE 2006 lens density spectrum at 400 nm and used the scaled densities to define the lens density spectrum from 400 to 360 nm. The assumed extension is shown by the solid black line in the middle panel of Figure 2.

The wavelength range for the lens template fit was 360 to 660 nm. Above 660 nm, the density is assumed to be zero. For *θ*_lens_ to vary from 0 to *π* from 360 to 660 nm:

(5) $$\theta_{\text{lens}}=\pi \frac{\lambda -360}{660-360}.$$

A 9th order Fourier polynomial captured the extended lens optical density spectrum extremely well. The fit is shown by the dashed white line in the middle panel of Figure 2, and the errors shown as the orange curve in the lower panel. The adjusted *R* ^2^ value is >99.99% and the standard error of the fit 0.004. The errors resulting from the use of this template rather than the original discrete tabulated functions are small, but slightly greater than the macular fit. This is partly because of the relatively abrupt slope change and flattening of the CIE 2012 template at and below 375 nm, and because of other minor discontinuities in the combined CIE 2006 and 2012 lens templates. The polynomial coefficients are given in Column 2 of Table 2. The lens density as a function of wavelength, $\text{lens}\left(\lambda \right)$, is given by substituting Equation (5) into Equation (3) with the appropriate values from Table 2.

As noted above, the main purpose of the short‐wavelength extensions was to facilitate spectral shifts of the L‐ and M‐cone spectra along a log wavelength axis. If any analyses are restricted to 390 nm and longer after shifting, the effects of errors in the short‐wavelength extrapolations should be small. Used with caution, the short‐wavelength extensions might be useful for estimating visual performance between 360 and 390 nm with the proviso that the lens template is likely to be too transparent for older observers.

### 2.3 From the continuous spectra to corneal cone fundamentals

By convention, absorbance spectra correspond to the spectral absorption of infinitely dilute photopigments (so that their shapes are independent of photopigment optical density).^41^ To calculate cone spectral sensitivities in the living eye, we need to know the axial optical density of the photoreceptors, which is proportional to the product of the concentration of the photopigments in situ and the length of the outer segment in which they reside and through which light passes.^41^ The length of the photoreceptor outer segment declines with eccentricity^42^, ^43^ and is shorter for S‐cones than for L‐ and M‐cones in the same retinal region.^44^ Thus, the photopigment optical density is lower for more peripheral lights and lower for S‐cones.

The peak photopigment optical densities assumed in the CIE standard for a 2‐deg field of view are 0.50, 0.50, and 0.40 for the L‐, M‐ and S‐cones, respectively; and, for a 10‐deg field of view, 0.38, 0.38 and 0.30 for the L‐, M‐ and S‐cones, respectively.^2^ Increasing the peak photopigment optical density broadens the spectral sensitivity of the cones by a process known as self‐screening.^45^ Thus, longer foveal cones will have the same *λ*_max_ as peripheral cones but be relatively more sensitive to wavelengths above and below the peak.

Using the CIE standard cone optical densities and the formulae for the cone absorbance spectra, we can calculate the spectral sensitivities of the cones without pre‐receptoral filtering, also known as the photopigment absorptance spectra.^41^ The spectral sensitivity of the unfiltered L‐cone photoreceptor, ${\bar{l}}_{R}\left(\lambda \right)$, is related to the L‐cone absorbance spectrum, ${\bar{l}}_{A}\left(\lambda \right)$ by:

(6a) $${\bar{l}}_{R}\left(\lambda \right)=1-10^{-l_{\mathrm{OD}}{\bar{l}}_{A}\left(\lambda \right)},$$

where *l*_OD_ is the peak L‐cone optical density, which, for the standard observer, is 0.5 for a 2‐deg field and 0.38 for a 10‐deg field. Similarly, for M and S*:

(6b) $${\bar{m}}_{R}\left(\lambda \right)=1-10^{-m_{\mathrm{OD}}{\bar{m}}_{A}\left(\lambda \right)},$$

(6c) $${\bar{s}}_{R}\left(\lambda \right)=1-10^{-s_{\mathrm{OD}}{\bar{s}}_{A}\left(\lambda \right)},$$

where *m*_OD_ is the peak M‐cone optical density, which for the standard observer is 0.5 for a 2‐deg field and 0.38 for a 10‐deg field; and *s*_OD_ is the peak S‐cone optical density, which for the standard observer is 0.4 for a 2‐deg field and 0.30 for a 10‐deg field.

To calculate from photoreceptor spectral sensitivities to corneal spectral sensitivities, ${\bar{l}}_{Q}\left(\lambda \right)$, the filtering by the lens and macular pigments is included:

(7) $${\bar{l}}_{Q}\left(\lambda \right)=\frac{{\bar{l}}_{R}\left(\lambda \right)}{10^{k_{\text{lens}}\text{lens}\left(\lambda \right)+k_{\mathrm{mac}}\mathrm{mac}\left(\lambda \right)}},$$

and similarly for M‐ and S‐cones. For the mean standard mean observer, *k*_mac_ is 1 for a 2‐deg field and 0.271 for a 10‐deg field, and *k*_lens_ is 1.0.

It is important to note that corneal cone spectral sensitivities calculated in this way are quantal spectral sensitivities, since they are related to quantal absorptions by the photopigment. It is more common in colorimetry to use energy units. As the energy of a photon is inversely proportional to its wavelength, to specify corneal spectral sensitivities in energy units, $\bar{l}\left(\lambda \right)$, we simply multiply by λ and renormalize to unity peak:

(8) $$\bar{l}\left(\lambda \right)=\alpha \lambda {\bar{l}}_{Q}\left(\lambda \right),$$

where α is a scaling constant that forces the function to peak at 1 (note that the change from quantal to energy units shifts λ_max_ slightly to longer wavelengths).

**FIGURE 3:**
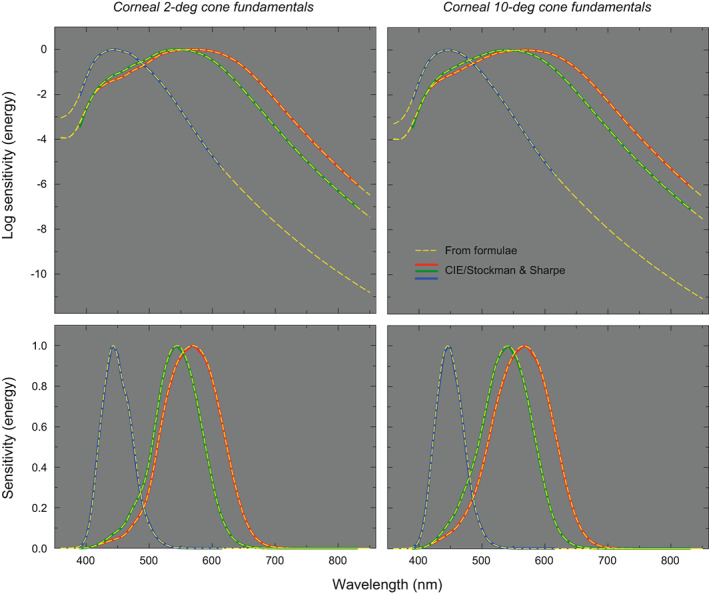
Logarithmic (upper panels) and linear (lower panels) corneal L‐, M‐ and S‐cone spectral sensitivities (yellow dashed lines) calculated from the Fourier polynomials for 2‐deg (left panels) and 10‐deg (right panels) vision. The CIE L‐, M‐ and S‐cone standards are shown by the red, green, and blue solid lines, respectively. The sensitivities are given in relative energy units.

Figure 3 shows the corneal L‐, M‐ and S‐cone spectral sensitivities generated from the formulae as the dashed yellow lines in four panels. The original CIE functions are shown by solid red, green and blue lines. The upper panels show logarithmic sensitivities and the lower panels the corresponding linear sensitivities. The left panels show the 2‐deg functions and the right panels the 10‐deg functions. The agreement between the tabulated and generated spectral sensitivities is excellent. Excluding *λ* < 400 nm, where the deviations are due to adjustments in the shapes of the absorbance spectra (see above), the mean absolute errors (MAEs) for points sampled at 1‐nm intervals are 0.0040 and 0.0018 for the 2‐deg logarithmic and linear fundamentals, respectively, and 0.0043 and 0.0020 for the 10‐deg logarithmic and linear fundamentals.

## 3 FORMULAE FOR NON‐STANDARD OBSERVERS AND FOR CHANGES WITH ECCENTRICITY

The CIE standards defined by the above equations and Fourier polynomial coefficients are useful for modeling color vision in those observers with normal cone spectral sensitivities and typical macular, lens, and photopigment optical densities. However, they are less useful for modeling color vision in observers whose spectral sensitivities deviate from the standard because of individual differences in the density of the lens pigment, the density of macular pigment, or the axial optical density of the photopigment in the photoreceptors. The utility of the formulae presented so far is that they can be used to generate cone fundamentals for individuals with different macular, lens and, photopigment optical densities. Macular pigment density can be varied by changing *k*_*mac*_ in Equation (7), lens density by changing *k*_lens_ in the same equation, and photopigment density by changing *l*_OD_, *m*_OD_ or *s*_OD_ in Equation (6).

Individual differences in lens pigment optical density can be large, particularly since lens density increases with the age of the observer.^46^, ^47^, ^48^ Yet, even in young observers of a similar age (<30 years old) the range of densities is approximately ±25% of the mean.^33^ Asano, Fairchild and Blondé^49^ usefully summarize the results of 13 past studies on the optical density of lens pigment in their Table 1. The CIE 2006 standard includes formulae and templates that change the shape of the lens pigment optical density spectrum with increasing age^5^ based on work by Pokorny, Smith and Lutze.^48^ These are not implemented here.

Individual differences in macular pigment optical density can also be large with a range of peak density from 0.0 to c.1.2 at 460 nm.^32^, ^50^, ^51^ The density of the pigment also changes with retinal location; tending to become more transparent with eccentricity, and becoming wholly or largely absent by a retinal eccentricity of 10 deg.^30^ Asano, Fairchild and Blondé^49^ usefully summarize the results of nine past studies on the optical density of macular pigment in their Table 2.

Cone photopigment optical density estimates depend on the method used to measure them but all show sizeable individual differences.^52^, ^53^, ^54^, ^55^, ^56^, ^57^, ^58^, ^59^ Measurements made before and after a bleach yield mean peak optical density values that range from 0.3 to 0.6, while those that depend on oblique presentation range from 0.7 to 1.0; and other objective measures range from 0.35 to 0.60. See Stockman and Sharpe^1^ for discussion. Asano, Fairchild and Blondé^49^ summarize the results of four past studies in their Table 3.

As well as differing among individuals, the macular pigment density and photopigment optical density vary with retinal eccentricity and must be adjusted when predicting the cone spectral sensitivities for target sizes and eccentricities different from the standard 2‐ and 10‐deg foveal viewing conditions. These can also be easily changed by changing *k*_mac_ in Equation (7) and changing *l*_OD_, *m*_OD_ or *s*_OD_ in Equation (6).

Variations in the spectral positions of the L‐ and M‐cone photopigments are common because of hybrid L‐ and M‐cone photopigment opsin genes. These hybrid genes are fusion genes produced by intragenic crossovers during meiosis between the L‐ and M‐cone opsin genes, which lie in a head‐to‐tail array on the X‐chromosome,^60^ and thus contain coding sequences from both genes; for review, see References [61, 62].

Both in vitro^63^, ^64^ and in vivo^7^, ^65^ determinations of the *λ*_max_ of the absorbance spectra of hybrid pigments reveal a wide range of possible anomalous pigments with peaks lying in steps between those of the normal L‐ and M‐cone pigments. In addition, smaller shifts occur within the normal population, because of different polymorphisms (commonly occurring allelic differences) of the M‐ and L‐cone photopigment opsin genes. The most frequently observed polymorphic‐induced shift occurs in the L‐cone photopigment when alanine replaces serine at position 180 of the L photopigment opsin gene, leading to a mean shift across studies of about 3.5 nm towards shorter wavelengths.^66^

The L‐ and M‐cone opsins are made up of a sequence of 364 amino acids, which are specified by “codons” (3‐base sequences of DNA nucleotides) “read” from the genes. Of these 364 amino acids, only 15 differ between the L‐ and M‐cone opsins, and of those only 7 are thought to change the spectral sensitivity of the photopigment.^61^ The seven L and M variants of the amino acids that alter spectral sensitivity are given in Table 3.

**TABLE 3:** Spectral shifts caused by changes in key amino acids in the L and M opsins.

Exon	Codon	M opsin amino acids	L opsin amino acids	Summary	
				M → L	L → M
2	116	Tyrosine	Serine	0	−3
3	180	Alanine	Serine	3	−4
4	230	Threonine	Isoleucine	3	−3
	233	Serine	Alanine	0	0
5	277	Phenylalanine	Tyrosine	7	−7
	285	Alanine	Threonine	14	−14
	309	Phenylalanine	Tyrosine	0	0

Changing the amino acids shown in Table 3 from the M to the L versions or vice versa, shifts the spectral sensitivity of the resulting photopigment. The shifts in Table 3 are based on data from various sources,^7^, ^62^, ^63^, ^64^, ^65^, ^67^ which are summarized in Table A1. The shifts are given in the two far right columns. Notice that the shifts are sometimes slightly asymmetric. Based on these earlier data, codons at positions 233 and 309 have been assumed not to shift the spectral peak. Thus, only 5 codons seem to be important for determining spectral sensitivity.

We have defined our absorbance spectra as functions of log wavelength so that, given the assumption that photopigment absorbance spectra are roughly shape invariant along such a scale,^19^, ^20^, ^21^ we can easily shift them along the spectrum to generate cone absorbance spectra with different *λ*_max_.

To calculate shifted absorbance functions, the spectral shift should be converted to *θ*_*P*_ units as defined in Equation (2). Spectral shifts, however, are usually given as linear wavelength shifts in nm at *λ*_max_ (i.e., as a shift from *λ*_max1_ to *λ*_max2_). The logarithmic shift is then log_10_(*λ*_max1_/*λ*_max2_) and the shift in *θ*_*P*_ units, Δ*θ*_*P*_, is then:

(9) $$\Delta \theta_{P}=\pi \frac{\log_{10}\left(\lambda_{\max1}/\lambda_{\max2}\right)}{\log_{10}\left(850/360\right)}.$$

Note that the L‐cone template given in Table 1 defines an L‐cone absorbance spectrum that is the mean of the polymorphic L(ala180) and L(ser180) variants in the population. In the next section, we define an L‐cone absorbance spectrum that is appropriate for individual L‐cone variants.

These formulae can also be used to spectrally shift the S‐cone absorbance spectrum, but unlike the M‐ and L‐cone spectra there is only weak evidence for such shifts. Estimating S‐cone *λ*_max_ from psychophysical measurements in five normal observers and three S‐cone monochromats, Stockman, Sharpe and Fach^22^ found that the mean *λ*_max_ across observers was 418.8 nm and the standard deviation was 1.5 nm (see their p. 2922). Given the uncertainties about pre‐receptoral filtering at shorter wavelengths, this variability is very small; however, Stockman et al. did note that their data fell roughly into two clusters with means of 417.4 and 420.1 nm.

The continuous spectral sensitivity functions generated by these formulae may be useful enhancements for the development of individual colorimetric observer models such as the one proposed by Asano, Fairchild and Blondé.^49^

## 4 FORMULA FOR AN L‐CONE POLYMORPHIC TEMPLATE

Two common L‐cone polymorphic variants are found in the normal population: one with alanine at position 180 of the opsin gene, L(ala180), and one with serine at position 180, L(ser180); for review, see Reference [61]. To generate the mean L‐cone fundamental, Stockman and Sharpe^2^ combined spectral sensitivity measurements from single‐gene dichromats with either L(ser180) or L(ala180), which they found differed in spectral position on average by 2.7 nm with L(ser180) shifted towards longer wavelengths.^7^ To produce the mean L‐cone spectral sensitivity subsequently adopted by the CIE,^5^, ^6^ Stockman and Sharpe^2^ linearly combined the L(ala180) and L(ser180) spectral sensitivities in the ratio of 0.44:0.56 (based upon the proportion of the polymorphisms found in the population).

Consequently, the L‐cone absorbance template given in Table 1 represents the weighted linear average of the absorbances of two L‐cone polymorphisms with slightly different spectral sensitivities. In this section, we derive a template shape that is appropriate for either the L(ser180) or the L(ala180) variants alone.

We repeated the fit to L carried out to obtain the L‐cone template given in Table 1, but assumed that the mean L‐cone absorbance spectrum was the linear addition of two identical templates combined in the ratio 0.44:0.56 with the one with the higher ratio shifted along the log wavelength scale by −0.002108 log_10_ nm (−2.7 nm from the 557.5‐nm *λ*_max_ of the best‐fitting L(ser180) template). As before, a simultaneous fit to both the linear and logarithmic absorbances was performed.

The adjusted *R* ^*2*^ value for the L(ala180) plus L(ser180) fit is >99.99%, and the standard error is 0.003. The fit and errors (not shown) are visually indistinguishable from the L‐cone fit and errors as shown in Figure 1. The *λ*_max_ of the L(ser180) function 553.1 nm and that of L(ala180) function 550.4 nm, which is a spectral shift of 2.7 nm or 0.002125 log_10_ nm. Column 2 of Table 4 gives the L(ser180) template. Shifting it by 0.002125 log_10_ nm to shorter wavelength gives the L(ala180) template.

**TABLE 4:** Fourier coefficients for the L(ser180) template optimized for L‐cone polymorphic spectra only (column 2) or for all cone spectra column 3).

Fourier coefficients	Polymorphic L, L(ser180)	Common LMS, L(ser180)
*a*_0_	−42.417609	−2.125656
*a*_1_	−2.656792	5.467793
*b*_1_	75.011094	0.896066
*a*_2_	56.477063	−0.953011
*b*_2_	7.509398	−5.037710
*a*_3_	9.061442	−3.003999
*b*_3_	−38.068488	−0.950862
*a*_4_	−20.974610	−1.367085
*b*_4_	−6.642746	1.770211
*a*_5_	−3.785039	0.516505
*b*_5_	9.322071	1.150550
*a*_6_	3.134495	0.610042
*b*_6_	1.603799	0.051821
*a*_7_	0.439302	0.100928
*b*_7_	−0.676959	−0.177357
*a*_8_	−0.072988	−0.027880
*b*_8_	−0.078858	−0.042774
*s*	−0.004264	0.000705

## 5 FORMULA FOR A COMMON SHAPE‐INVARIANT PHOTOPIGMENT TEMPLATE

The reason for separately constructing Fourier polynomials for the absorbance spectrum of each of the three cone types is because the underlying CIE spectra have slightly different shapes even when plotted against a logarithmic function of wavelength. Thus, to compute continuous CIE cone fundamentals and minimize the differences from the tabulated spectra, color matching functions, and chromaticity coordinates, we needed to generate separate photopigment absorbance functions for each cone type.

Here, we generate an optimal *common* photopigment absorbance spectrum that best fits all three CIE cone absorbance spectra. Such a template helps to highlight the differences in shape between the CIE spectra but is also useful for generating absorbance spectra for photopigments in other species.

As in the previous section, we assumed that the CIE L‐cone absorbance spectrum is a linear combination of the underlying L(ala180) and L(ser180) spectra in the ratio 0.44:0.56. We derived the best‐fitting common template by aligning it with the L(ser180) spectrum and then allowing the template to shift along a log wavelength scale for other spectra. The shift to L(ala180) was fixed at −0.002108 log_10_ nm (−2.7 nm from the 557.5‐nm *λ*_max_ of the best‐fitting L(ser180) template consistent with the shift adopted by Stockman and Sharpe), while the shifts to M and S were best‐fitting shifts. As before, a simultaneous fit to both the linear and logarithmic absorbances was performed, and the extension of the S‐cone absorbance function between 360 and 390 nm was not allowed to influence the fit since it had already been averaged from the L‐ and M‐cone templates (see above).

**FIGURE 4:**
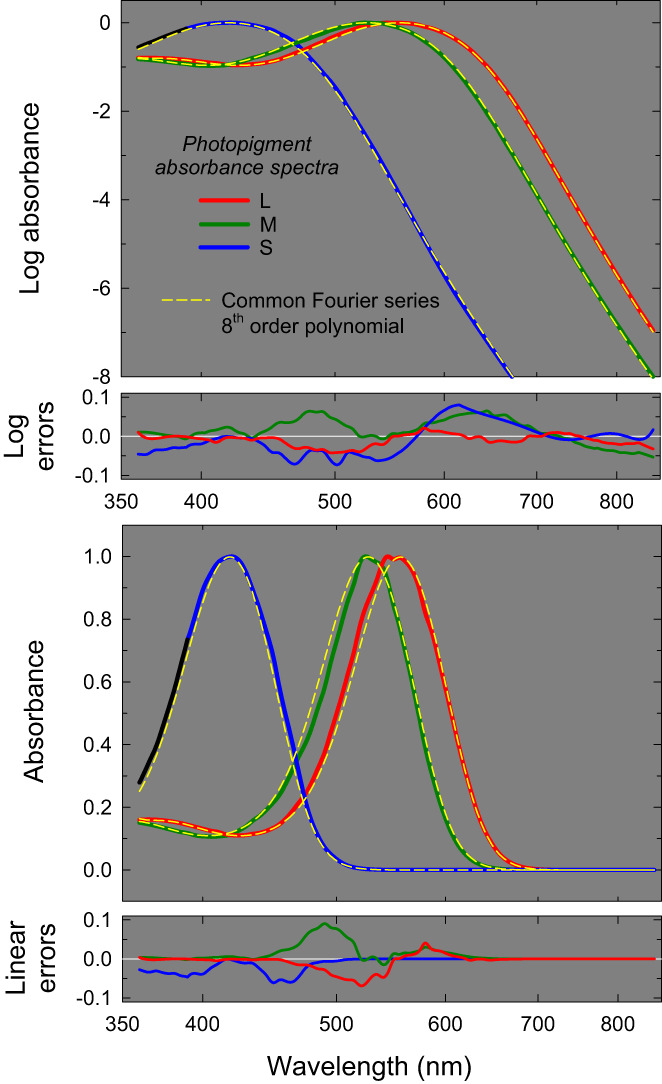
The extended logarithmic (top panel) and linear (third panel) L‐, M‐ and S‐cone photopigment absorbance spectra (red, green, and blue solid lines, respectively) fitted by the same 8th order Fourier polynomial (yellow dashed lines) shifted along the log‐wavelength scale. The L‐cone spectrum was assumed to be a linear combination of two underlying common spectra: one for L(ala180) and the other for L(ser180). The black portion of the S‐cone spectrum was not included in the fit. See text for further details.

Figure 4 shows again the extended CIE absorbance spectra used to derive the Fourier polynomial as the solid red, green and blue lines for L‐, M‐ and S‐cones, respectively, plotted as logarithmic absorbances in the top panel and linear absorbances in the third panel; wavelength is plotted on a logarithmic scale in all panels. The regions of the S‐cone functions not included in the fit are shown in black. The best‐fitting shifted common template that fits the CIE functions are shown by the three dashed yellow lines (the L‐cone fit is, of course, the weighted sum of L(ser180) and L(ala180)). The Fourier polynomial accounts reasonably well for the three cone absorbances on both logarithmic and linear scales. The logarithmic errors are shown in the second panel of Figure 4 and the linear errors in the bottom panel. The errors are much larger than those found for individually fitted templates (see Figure 1 and note the change of scale in the error plots) and reflect mainly differences in the widths of the underlying CIE absorbance spectra that cannot be accounted for by a single template shape. Nevertheless, the adjusted *R* ^*2*^ value for the fit is >99.99% and the standard error of the fit is 0.0260. However, the fit with mixed L(ala180) and L(ser180) templates is no better than the fit with a single template (not shown), and both are considerably worse than the individual template fits.

Column 2 of Table 4 gives the coefficients that define the L(ser180)‐cone spectrum without any spectral shift. Relative to this spectrum the L(ala180)‐spectrum should be shifted by −0.002108 log nm (consistent with the assumed 2.7‐nm shift), the M‐cone spectrum by −0.024187 log nm, and the S‐cone spectra by −0.124549 log nm. These correspond to shifts in the *λ*_max_ of the common L(ser180) photopigment absorbance spectrum from its peak at 557.5 (its unshifted value) to 554.8 nm for L(ala180) to 527.3 nm for M and to 418.5 nm for S. The peak of the combined L(ser180) and L(ala180) template is 556.0 nm.

For the common template the log wavelength scale is equivalent to a “normalized frequency” scale (i.e., *f*/*f*_max_ or *λ*_max_/*λ*, since *f* is proportional to 1/*λ*). Shape invariance along such a scale can thus be linked to relative quantal sensitivity^19^, ^69^ (since the energy of a photon is proportional to its frequency). Nonetheless, the fits shown in Figure 4, particularly the linear fit in the third panel, suggest that shape invariance along a log wavelength scale is at best approximate, since the M‐cone absorbance function is clearly narrower than either the S‐cone or L‐cone absorbances. There are two plausible reasons why the M‐cone function might be narrower than the L‐cone function. First the M‐cone photopigment optical density might be less than the L‐cone density rather than being equal to it as previously assumed.^2^ Indeed, several groups have found that the M‐cone optical density is lower than that of L‐cones by about 0.1 to 0.2 log unit, but the sample sizes in those studies were small and other evidence is contradictory; for review see Reference [1]. Furthermore, a more recent study with large sample sizes of 28 protanopes and 44 deuteranopes showed no difference between the M‐ and L‐cone optical densities.^70^ Nevertheless, a lower M‐cone optical density could account for some of the differences in shape that we find. Similarly, if the S‐cone photopigment density is considerably higher than that of M‐cones this could account for the different widths of their absorbance functions, but this is unlikely because, as noted above, the available evidence is clear that S‐cone density is in fact lower than M‐cone. A second reason that the M‐cone function might be narrower than the L‐cone one is that the L‐cone function is the population mean of two polymorphic variants of the L‐cone photopigment (one with serine at position 180 and another with serine at position 180) with *λ*_max_ values that differ by 2.7 nm (see above). However, this assumption was explicitly made when we fitted the spectra shown in Figure 4 to a common template and was insufficient to account for the shape differences (it would also not account for the M‐cone's absorbance spectrum being narrower than that of the S‐cone).

## 6 CONE SPECTRAL SENSITIVITIES AND COLORIMETRY

In this section, we relate the cone spectral sensitivities to colorimetry and color matching. The formulae we have derived to generate the cone fundamentals can be straightforwardly used to construct other color matching functions and chromaticity coordinates. However, to help explain these procedures, we first introduce some of the concepts and nomenclature used in colorimetry.

**FIGURE 5:**
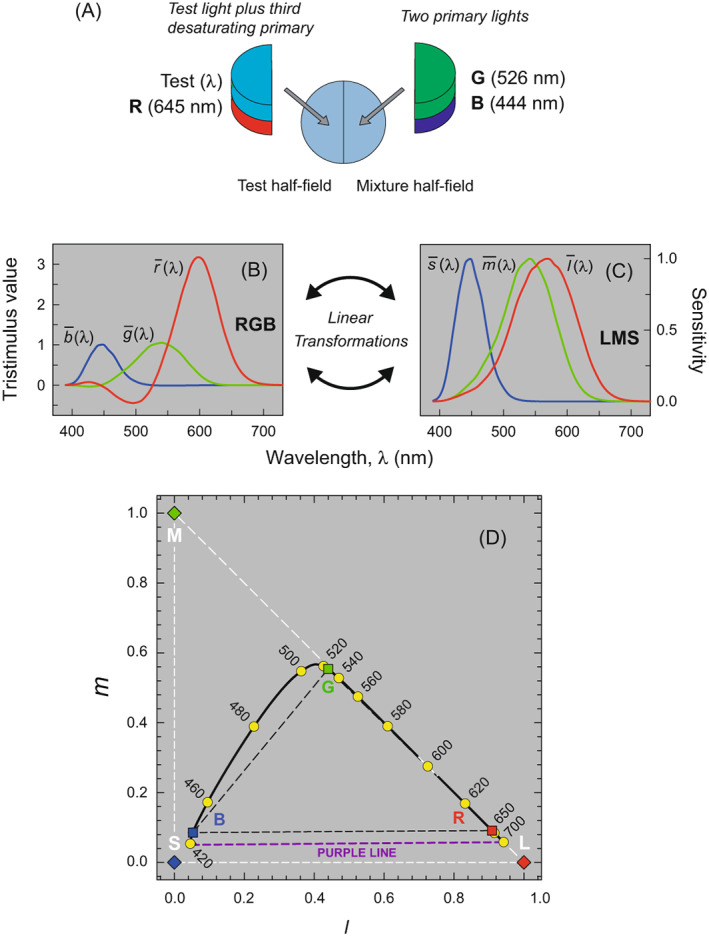
(A) Maximum saturation method of setting a color match. A monochromatic test field of wavelength, *λ*, can be matched by a mixture of red (645 nm), green (526 nm) and blue (444 nm) primary lights, one of which must be added to the test field to complete the match. In this example, the red primary must be added to match a cyan (c. 490 nm) test light. (B) The amounts of each of the three primaries required to match monochromatic lights spanning the visible spectrum are the $\bar{r}\left(\lambda \right)$, $\bar{g}\left(\lambda \right)$, $\bar{b}\left(\lambda \right)$ CMFs. (C) Color matches produce identical quantum catches in the three cone types. Thus, the cone fundamentals CMFs $\bar{l}\left(\lambda \right)$, $\bar{m}\left(\lambda \right)$, and $\bar{s}\left(\lambda \right)$ (or the cone spectral sensitivities) must be a linear transformation of the $\bar{r}\left(\lambda \right)$, $\bar{g}\left(\lambda \right)$, $\bar{b}\left(\lambda \right)$ CMFs. (D) The *l,m* cone chromaticity plane with the coordinates of the spectrum locus shown by the solid line and yellow circles. The purple line joins the bottommost points on the locus and is the limit of physically realizable colors in the violet to red region of the chromaticity space (dashed purple line). The red, green, and blue diamonds are the intersections of the **L**, **M** and **S** primary vectors with the *l,m* cone chromaticity plane. The white dashed lines that join them delimit the imaginary and real colors that can be matched by adding together those primaries. The red, green, and blue squares are the intersections of the vectors representing the **R**, **G** and **B** primaries with this plane. The blacked dashed lines joining them show the real colors that can be matched by adding together those primaries. The figure shows 10‐deg color matching data.

In colorimetry, color is characterized in terms of how it can be matched to a mixture of three lights called “primary lights”. A typical matching experiment is illustrated in Panel (A) of Figure 5. The primary lights in this example are the 444 (**B**), 526 (**G**) and 645 (**R**) nm lights used by Stiles and Burch^71^ for their 10‐deg color matching measurements (note that primaries are usually referred to by bold capital letters). In theory any three lights can be used as primaries, so long as no primary can be matched to a combination of the other two. The test lights in this experiment are monochromatic lights of wavelength, *λ*. Observers were asked to match a test light of wavelength, *λ*, in one half of a 10‐deg field with a mixture of the three primaries, and the matches were made as a function of test wavelength to produce the color matching functions (CMFs), denoted $\bar{r}\left(\lambda \right)$, $\bar{g}\left(\lambda \right)$, and $\bar{b}\left(\lambda \right)$, shown in Panel (B) (note that CMFs are denoted by lower case italics as functions of wavelength). These functions give the amounts of the three primary lights required to match test lights of equal energy across the spectrum.^†^ The amounts of primary lights are known as the tristimulus values, which are denoted by upper case italics (e.g., *R*, *G*, and *B*). Except at the primary wavelengths, one of the Stiles and Burch CMFs is always negative. Below 444 nm and above 645 nm $\bar{g}\left(\lambda \right)$ is negative, between 444 and 526 nm $\bar{r}\left(\lambda \right)$ is negative, and between 526 and 645 nm $\bar{b}\left(\lambda \right)$ is negative. Mathematically, a negative CMF value for a given primary means that that primary must be subtracted from the other two primaries to complete the match to the test. In practical terms, since lights cannot be subtracted in this way (negative light is not physically realizable), the negative sign means that the primary light has been added to the test light to complete the match against the other two primaries. This is shown in Panel (A) where a bluish test light (*λ* ≈ 490 nm, and $\bar{r}\left(\lambda \right)<0$) requires the red primary to be added to the test to match a combination of the blue and green primaries. When added to the test light, the primary “desaturates” it.

In these kinds of experiments in which the two fields are presented in the same context, a color match is made at the level of the cones in that when a match is made, the excitations of the three cone types produced by the lights making up each of the matching semi‐circular fields should be identical (note, this is different from *asymmetric* color matches where the matched lights are shown in different contexts (e.g., under different illuminations) and can produce different cone excitations but give rise to the same color sensation, or vice versa). A consequence of color matching being determined at the level of the cones is that the color matching functions, $\bar{r}\left(\lambda \right)$, $\bar{g}\left(\lambda \right)$, and $\bar{b}\left(\lambda \right)$, in Panel (B) of Figure 5 (or any other set of CMFs measured using any three primaries) must be a linear transformation of the three 10‐deg CIE cone fundamental CMFs shown in Panel (C) and vice versa (this relation is formalized by Grassmann's laws of color matches^72^; a helpful version of which in terms of matching symmetry, transitivity, proportionality, and additivity is given by Wyszecki and Stiles^73^ p. 118) Note that the usual colorimetric nomenclature for CMFs (e.g., $\bar{r}\left(\lambda \right)$, $\bar{g}\left(\lambda \right)$, $\bar{b}\left(\lambda \right)$) can also be used for cone fundamentals ($\bar{l}\left(\lambda \right)$, $\bar{m}\left(\lambda \right)$, and $\bar{s}\left(\lambda \right)$). The cone spectral sensitivities are thus the “fundamental” color matching functions or cone fundamental CMFs on which all other CMFs depend.

To help explain the relation between the primaries and their color matching functions, as well as the relation between **RGB** and **LMS** CMFs, we have plotted in Panel (D) of Figure 5 the primaries and the spectrum locus in a 2D plot, using what are known as chromaticity coordinates. The three cone spectral sensitivities or cone fundamentals allow colors and lights to be straightforwardly defined within a three‐dimensional vector space corresponding to the triplet of cone excitations each produces. Any such color or light can be described by its tristimulus coordinates (*L*, *M*, *S*). Note, for an arbitrary real light **P** with power spectrum *P*(*λ*), the tristimulus values are defined as, for example., $L=\int P\left(\lambda \right)\bar{l}\left(\lambda \right)d\lambda$, and similarly for M‐ and S‐cones. A given tristimulus value thus corresponds to all lights that would produce the same three cone responses that is, all possible metamers. However, it is often helpful for visualization to project this 3‐dimensional vector space into a 2‐dimensional plane. The projective dimension is typically either luminance or intensity, so that what remains is chromaticity. One such projection plots all points along a line through the origin onto the plane with the equation:

(10a) L+M+S=1.

The *l,m* chromaticity diagram is a projective transformation of this plane, such that the *l* chromaticity coordinate is:

(10b) $$l=\frac{L}{L+M+S},$$

and the *m* chromaticity coordinate is:

(10c) $$m=\frac{M}{L+M+S}.$$

The *s* chromaticity coordinate, which is *s* = 1 − (*l* + *m*), is not plotted. Although the *l,m* representation may be unfamiliar, we prefer it as it is the only one that corresponds to the lowest level of vision—the cone activations—unlike say MacLeod‐Boynton space,^74^ which is supposed to correspond to cone‐opponent processes in the early visual pathways, or the hue and chroma coordinates of the Munsell color system,^75^ which are linked to color appearance.

Panel (D) of Figure 5 shows the fundamental CMFs plotted in *l,m* chromaticity coordinates. The locus of the spectrum is shown as the solid black line with selected wavelengths highlighted by the yellow circles. This locus and the purple line joining its bottommost points contains the area within which colors can be produced by real lights or by mixtures of real (physically realizable) lights. Points outside this area cannot be produced by real lights. The **R**, **G** and **B** primaries of Stiles and Burch are shown as colored squares and the black dashed triangle encloses all the colors that can be matched to positive amounts of **R**, **G** and **B** that is, the color gamut of these primaries. The three primary lights of 444, 526 and 645 nm chosen by Stiles and Burch enclose a large area of the realizable color gamut delimited by the spectrum locus and purple line shown in Panel (D). Although the spectrum locus lies close to the line connecting the **G** and **R** primaries in this plot, it in fact lies just outside the gamut (except at its corners) so that as previously mentioned one of the CMFs must be negative at all wavelengths. Also shown are the three **LMS** cone primaries as color diamonds connected by dashed white lines; these lie outside the gamut not only of **RGB** but also of the spectrum locus so that they are *imaginary* lights. The cone primaries are lights which, if they could be produced, would uniquely stimulate a single cone type. They cannot be produced because the cone spectral sensitivities overlap extensively throughout the spectrum, so that all lights excite more than one type. Note that the dashed white triangle fully encloses the spectrum locus so that the **LMS** CMFs in Panel (C) are everywhere positive as would be expected given the fundamental nature of the cone activations.

Two caveats are perhaps of interest here. First, the unique *modulation* of a single cone type is possible if the light is modulated around a non‐zero mean, since this allows modulations both above and below the non‐zero mean by a process called “silent substitution”.^76^ Yet since the mean light also stimulates the other two cones the activation is not unique—only the modulation is. Second, with adaptive optics and single cone stimulation it is possible to excite single cones or multiple cones of a single type, so that it is possible to produce colors that would be produced by imaginary lights outside the spectrum locus.^77^, ^78^, ^79^ It remains an open question how those colors are perceived.

In summary, the imaginary **LMS** primaries enclose the spectrum locus and produce CMFs that are always positive. Other imaginary primaries, such as **XYZ**, see below, can be chosen that also enclose the locus so that their CMFs are always positive. Real primaries, such as **RGB**, must lie on or within the spectrum locus (plus the purple line) and produce CMFs at least one of which is non‐positive at each wavelength.

## 7 LINEAR TRANSFORMATIONS BETWEEN COLOR‐MATCHING SPACES

The **LMS** space is the fundamental, physiologically relevant color‐matching space since, in principle at least, it reflects the relative excitations of cones themselves. Nevertheless, many prefer to plot color matches in other spaces. We consider two such color‐matching spaces here: an **RGB** space with physically realizable primaries, and the **XYZ** space with the imaginary primaries introduced by the CIE in their 1931 proceedings^80^ and consequently used extensively in applied color fields and in colorimetric devices.

To be consistent with Grassmann's laws,^26^, ^81^ any color‐matching space must be a linear transformation of the **LMS** space, and consequently any two color‐matching spaces must be linear transforms of each other. Converting between spaces simply requires specifying the relevant linear transformation—usually in the form of the components of a 3 × 3 matrix.

The linear transformation from **RGB** space to **LMS** space is given by:

(11a) $$\left(\begin{array}{ccc} L_{R} & L_{G} & L_{B}\\ M_{R} & M_{G} & M_{B}\\ S_{R} & S_{G} & S_{B} \end{array}\right)\left(\begin{array}{c} R\\ G\\ B \end{array}\right)\;=\;\left(\begin{array}{c} L\\ M\\ S \end{array}\right).$$

The nine elements of this matrix are the three cone spectral sensitivities (in energy units) to each of the three physical primaries. For example, for a monochromatic light of wavelength $\lambda_{R}$, $L_{R}=\bar{l}\left(\lambda_{R}\right)$ is the sensitivity of the L‐cones to **R**. Similar definitions apply to the other elements of the 3 × 3 matrix in relation to the other primaries and cone types (note that for broadband primary lights the sensitivities are replaced by, for example, $L_{R}=\int R\left(\lambda \right)\bar{l}\left(\lambda \right)d\lambda$, where $R\left(\lambda \right)$ is the power spectrum of the red primary [it is to avoid this complication that monochromatic lights are chosen]) Equation (11a) allows us to convert from an **RGB** color‐matching space to **LMS** space by breaking down each of the real **RGB** lights into their separate effects on the L‐, M‐ and S‐cones, and adding them together.

As a specific case of Equation (11a) we can transform **RGB** CMFs into **LMS** CMFs,

(11b) $$\left(\begin{array}{ccc} L_{R} & L_{G} & L_{B}\\ M_{R} & M_{G} & M_{B}\\ S_{R} & S_{G} & S_{B} \end{array}\right)\left(\begin{array}{c} \bar{r}\left(\lambda \right)\\ \bar{g}\left(\lambda \right)\\ \bar{b}\left(\lambda \right) \end{array}\right)\;=\;\left(\begin{array}{c} \bar{l}\left(\lambda \right)\\ \bar{m}\left(\lambda \right)\\ \bar{s}\left(\lambda \right) \end{array}\right).$$

Indeed, this is how the CIE cone spectral sensitivities are defined: as a linear transformation of the 10‐deg **RGB** CMFs of Stiles and Burch,^71^ since of the available sets of directly measured CMFs, they are the most secure and most comprehensive. The transformation from the Stiles and Burch 10‐deg ${\bar{r}}_{10}\left(\lambda \right)$, ${\bar{g}}_{10}\left(\lambda \right)$, and ${\bar{b}}_{10}\left(\lambda \right)$ CMFs to the three 10‐deg cone fundamentals, ${\bar{l}}_{10}\left(\lambda \right)$, ${\bar{m}}_{10}\left(\lambda \right)$, and ${\bar{s}}_{10}\left(\lambda \right)$, is^5^:

(12) $$\left(\begin{array}{ccc} 2.846201 & 11.09240 & 1\\ 0.168926 & 8.265895 & 1\\ 0 & 0.010600 & 1 \end{array}\right)\left(\begin{array}{c} {\bar{r}}_{10}\left(\lambda \right)\\ {\bar{g}}_{10}\left(\lambda \right)\\ {\bar{b}}_{10}\left(\lambda \right) \end{array}\right)=\left(\begin{array}{c} {\bar{l}}_{10}\left(\lambda \right)\\ {\bar{m}}_{10}\left(\lambda \right)\\ {\bar{s}}_{10}\left(\lambda \right) \end{array}\right).$$

The coefficients of the 3 × 3 matrix are the cone spectral sensitivities to the **RGB** primaries, which were determined from an extensive set of spectral sensitivity measurements and color matches made in genotyped color deficient observers, some normal observers and from the 10‐deg matches themselves.^7^, ^22^, ^27^, ^82^ The standard 2‐deg cone spectral sensitivities are based on the same transformation, but were adjusted for macular and photopigment optical densities appropriate for a 2‐deg target field; for details see Reference [2]. Note that the units of cone activation are somewhat arbitrary, since the absolute values are unknown, so there are scaling factors for each cone in converting from **RGB** to **LMS** and these scaling factors have been absorbed into the blue coefficients, so they are normalized to 1 (see third column of the 3 × 3 matrix in Equation (12)). The bottom left coefficient, $S_{R}$ in Equation (12), indicates the response of the S‐cones to **R**, and is assumed to be zero (this will be true for any **R** unless it is of unusually short wavelength).

Given that we know and have generated $\bar{l}\left(\lambda \right)$, $\bar{m}\left(\lambda \right)$, and $\bar{s}\left(\lambda \right)$ using the formulae above, how do we linearly transform those CMFs to a new set of real primaries? And more generally, how do we convert *LMS* space into some other *RGB* space? The procedure is straightforward. First, we populate the coefficients of the upper 3 × 3 matrix in Equation (11a) (i.e., input the cone spectral sensitivities to the new **RGB** primaries). Then we find the inverse matrix and calculate the second row of Equation (13):

(13) $$\begin{array}{c} \;\left(\begin{array}{c} R\\ G\\ B \end{array}\right)={\left(\begin{array}{ccc} L_{R} & L_{G} & L_{B}\\ M_{R} & M_{G} & M_{B}\\ S_{R} & S_{G} & S_{B} \end{array}\right)}^{-1}\left(\begin{array}{c} L\\ M\\ S \end{array}\right)\;\\ \left(\begin{array}{c} R\\ G\\ B \end{array}\right)=\left(\begin{array}{ccc} R_{L} & R_{M} & R_{S}\\ G_{L} & G_{M} & G_{S}\\ B_{L} & B_{M} & B_{S} \end{array}\right)\left(\begin{array}{c} L\\ M\\ S \end{array}\right)\; \end{array}.$$

In theory, the coefficients of the lower 3 × 3 inverse matrix are the tristimulus values produced by the three imaginary cone primaries, e.g., $R_{L}=\int L\left(\lambda \right)\bar{r}\left(\lambda \right)d\lambda$, where $L\left(\lambda \right)$ is the imaginary spectral power distribution of the L‐cone primary. As in Equations (11b) and (12) we can substitute CMFs in place of the generic tristimulus values in Equation (13) to convert the **LMS** CMFs into **RGB** CMFs.

As well as CMFs of real primaries, $\bar{l}\left(\lambda \right)$, $\bar{m}\left(\lambda \right)$, and $\bar{s}\left(\lambda \right)$ can be linearly transformed to CMFs for imaginary primaries such as the familiar colorimetric variants: $\bar{x}\left(\lambda \right)$, $\bar{y}\left(\lambda \right)$, and $\bar{z}\left(\lambda \right)$—a form still in common use in applied areas of research—by making a few simple assumptions.^6^ First, the $\bar{y}\left(\lambda \right)$ and ${\bar{y}}_{10}\left(\lambda \right)$ CMFs are assumed to be linear combinations of $\bar{l}\left(\lambda \right)$ and $\bar{m}\left(\lambda \right)$ or ${\bar{l}}_{10}\left(\lambda \right)$ and ${\bar{m}}_{10}\left(\lambda \right)$, respectively, defined by the second row of the 3 × 3 matrices in Equations (14) and (15) below. Second, the $\bar{z}\left(\lambda \right)$ and ${\bar{z}}_{10}\left(\lambda \right)$ CMFs are simply scaled versions of $\bar{s}\left(\lambda \right)$ and ${\bar{s}}_{10}\left(\lambda \right)$, respectively, defined by the third row of the 3 × 3 matrices. Last, $\bar{x}\left(\lambda \right)$ and ${\bar{x}}_{10}\left(\lambda \right)$ were chosen for consistency with the earlier CIE CMFs. See the CIE publication for further details.^6^

The 2‐deg transformation from the **LMS** to **XYZ** CMFs is given by:

(14) $$\left(\begin{array}{ccc} 1.94735469 & -1.41445123 & 0.36476327\\ 0.68990272 & 0.34832189 & 0\\ 0 & 0 & 1.93485343 \end{array}\right)\left(\begin{array}{c} \bar{l}\left(\lambda \right)\\ \bar{m}\left(\lambda \right)\\ \bar{s}\left(\lambda \right) \end{array}\right)=\left(\begin{array}{c} \bar{x}\left(\lambda \right)\\ \bar{y}\left(\lambda \right)\\ \bar{z}\left(\lambda \right) \end{array}\right),$$

where $\bar{l}\left(\lambda \right)$, $\bar{m}\left(\lambda \right)$, and $\bar{s}\left(\lambda \right)$ are the CIE 2006 2‐deg cone fundamentals and the 10‐deg transformation is given by:

(15) $$\left(\begin{array}{ccc} 1.93986443 & -1.34664359 & 0.43044935\\ 0.69283932 & 0.34967567 & 0\\ 0 & 0 & 2.14687945 \end{array}\right)\left(\begin{array}{c} {\bar{l}}_{10}\left(\lambda \right)\\ {\bar{m}}_{10}\left(\lambda \right)\\ {\bar{s}}_{10}\left(\lambda \right) \end{array}\right)=\left(\begin{array}{c} {\bar{x}}_{10}\left(\lambda \right)\\ {\bar{y}}_{10}\left(\lambda \right)\\ {\bar{z}}_{10}\left(\lambda \right) \end{array}\right),$$

where ${\bar{l}}_{10}\left(\lambda \right)$, ${\bar{m}}_{10}\left(\lambda \right)$, and ${\bar{s}}_{10}\left(\lambda \right)$ are the CIE 2006 10‐deg cone fundamentals.

**FIGURE 6:**
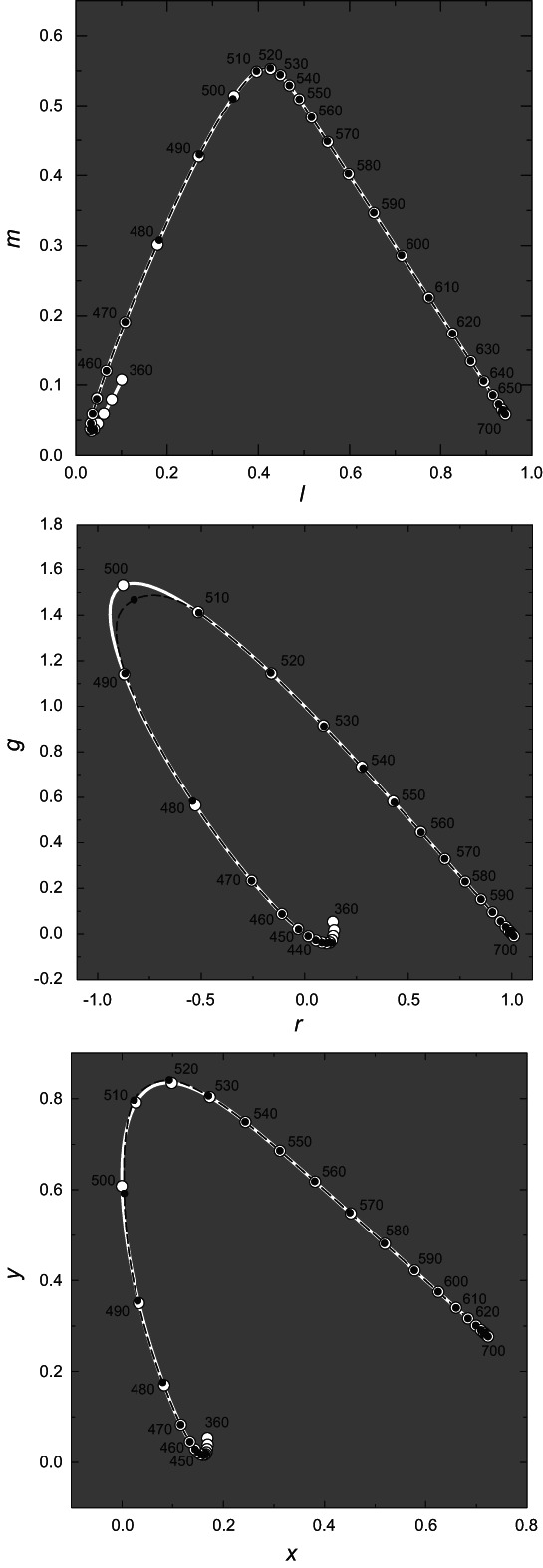
Cone fundamentals transformed to other primaries and plotted as chromaticity coordinates calculated from the original tabulated CIE 2006 fundamentals (dashed black lines, and small black circles at 10‐nm steps) and from the formulae presented here (solid white lines, and larger white circles). Upper panel: spectrum locus in *l,m* chromaticity coordinates. Middle panel: spectrum locus in *r*,*g*, chromaticity coordinates for RGB primaries of 444, 526 and 645 nm. Lower panel: spectrum locus in *x*,*y*, chromaticity coordinates for CIE XYZ primaries.

Using these transformations, we plot in Figure 6 three chromaticity diagrams each showing our cone fundamentals as the spectrum locus (as white curves and white circles) and the CIE 2006 10‐deg cone CMFs (as black dashed curves and black circles). The upper panel shows the *l,m* coordinates described above. The middle panel shows the spectrum locus in *r*,*g* chromaticity coordinates for $\bar{l}\left(\lambda \right)$, $\bar{m}\left(\lambda \right)$, and $\bar{s}\left(\lambda \right)$ transformed to the Stiles and Burch **RGB** primaries of 444, 526 and 645 nm, and the lower panel shows the spectrum locus in *x*,*y*, chromaticity coordinates for $\bar{l}\left(\lambda \right)$, $\bar{m}\left(\lambda \right)$, and $\bar{s}\left(\lambda \right)$ transformed to the **XYZ** primaries according to Equation (15). Note, *r,g* and *x,y* coordinates are derived from *RGB* and *XYZ* tristimulus values in the same way *l,m* are derived from *LMS* that is, $r=R/\left(R+G+B\right)$, $g=G/\left(R+G+B\right)$, $x=X/\left(X+Y+Z\right)$, and $y=Y/\left(X+Y+Z\right)$.

The agreement is excellent except for a minor discrepancy centered at 500 nm in *r,g* coordinates in the middle panel. This discrepancy reflects mainly the exaggerated stretching of this space (compared with *l,m* and *x,y* diagrams), and is likely to have little practical effect on color matching predictions.^83^ The agreement between the spectral loci transformed from the tabulated and generated CIE cone fundamentals is excellent for both *l,m* and *x*,*y* with mean absolute errors (MAEs) for points sampled at 1‐nm intervals of 0.0006 for *l*(*λ*), 0.0008 for *m*(*λ*), 0.0007 for *x*(*λ*) and 0.0011 for *y*(*λ*) but because of the discrepancy around 500 nm the agreement is less good for *r*,*g* with MAEs of 0.0025 for *r*(*λ*) and 0.0029 for *g*(*λ*).

In general, the formulae presented here can be used to generate CMFs and chromaticity coordinates for any arbitrary set primaries with small errors compared to the tabulated CIE data.

## 8 COLOR VISION IN THE UVA REGION ABOVE 360 NM

**FIGURE 7:**
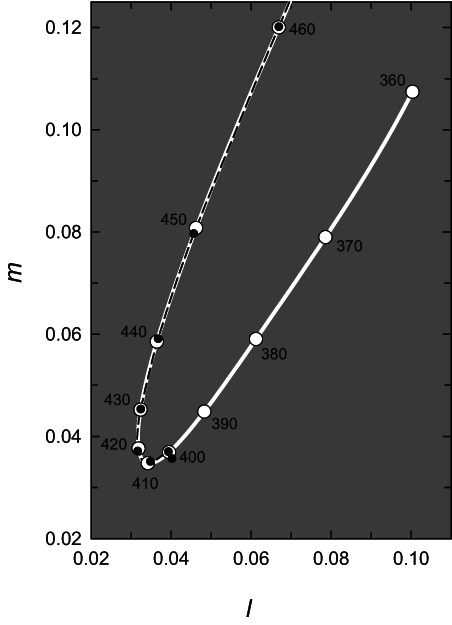
An expanded view of the short‐wavelength region of the spectrum locus in *l,m* coordinates. Symbols as in Figure 6.

The extrapolations of the three cone absorbance spectra from 400 to 360 nm predict the relative cone excitations and thus color matches in that spectral region. The predictions can be seen clearly in the three panels of Figure 6, or blown up in *l,m* coordinates in Figure 7. As wavelength decreases below 420 nm, the spectrum locus reverses and then moves diagonally up and towards the right reflecting slight increases in both the L‐ and the M‐cone spectral sensitivities in that region resulting from *β*‐band absorbances, and a corresponding decrease in S‐cone sensitivity (see Figure 1). Thus, lights would be expected to appear less violet (i.e., bluer) and more desaturated as the wavelength decreases in this short wavelength region. Yet, how plausible, or indeed useful are these color matching predictions?

Expectations of a rotation in the spectrum locus and a color reversal in long‐wavelength UVA region due to *β*‐band cone absorbances have been stated before,^84^, ^85^ but are those expectations consistent with the color appearances of those lights? Helmholtz^86^ noted that the violet color of 400 nm turned back towards blue as the wavelength decreased, which is consistent with the chromaticity diagram of Figure 7. However, color perception in this spectral region, as Helmholtz pointed out, is complicated by the fluorescence of the lens and retina. Lenticular fluorescence in the UVA region is primarily due to a fluorophore with an excitation maximum at 360 nm that emits light with an emission maximum between 420 and 440 nm and therefore alters the color appearance of the exciting light; see, for reviews.^87^, ^88^

The effects of lenticular fluorescence can be eliminated, and UVA sensitivity increased, by using aphakic observers who lack the lens. In such observers, Gaydon^89^ and Bachem^90^ reported that shorter wavelengths look bluer, which again is consistent with the color reversal predicted in the violet corner of the chromaticity diagram of Figure 7. Tan^91^ made more extensive color matching measurements in two aphakic observers, the mean results from which are replotted as chromaticity coordinates in figure 8 of Stark and Tan.^85^ The mean spectrum locus for these two aphakic observers, like that shown in Figure 7, reverses in in the violet part of the spectrum and remains broadly consistent with those predictions until about 370 nm. After 370 nm, surprisingly, the spectral locus of these aphakic observers continues to rotate until it points towards 445 nm. This further rotation is indicative of a relative increase in S‐cone excitation in that region. Tan points out on p. 78 of his thesis, however, that because of system limitations, “The results must therefore be judged qualitatively (rather) than quantitatively”, so it is not clear how much we can rely on these color matching data. Importantly, though, as Tan also points out the finding that UV lights can be matched using three primaries means that there is no evidence for another unknown photopigment in the UV.^91^ Yet other evidence from one of the two aphakic observers who made the color matches obtained using chromatic adaptation to favor different cone responses suggested that the S‐cone sensitivity steadily *increases* in the UVA implausibly rising above its sensitivity at the S‐cone *λ*_max,_ ^91^ a finding later replicated in another aphakic observer.^92^ An increase in S‐cone sensitivity into the UVA would account for the continued rotation of the aphakic spectrum locus below 370 nm, but its cause is unknown; see, for discussion,^85^ and it is inconsistent with known human and primate S‐cone photopigment spectra.^24^

In general, the color matching predictions in the UVA shown in Figures 6 and 7 are plausible but their usefulness in predicting color matches is inevitably limited, since any matches will be affected by fluorescence and perhaps by other unknown factors. New measurements in the UVA are undoubtedly needed, but to be useful the effects of fluorescence and cone spectral sensitivity must be disentangled.

## 9 CONCLUSION

A practical set of formulae is presented that allows the generation of cone fundamentals for standard (mean) observers for 2‐deg and 10‐deg vision that accurately reproduce the CIE 2006 observer. These fundamentals have been extended from 390 to 360 nm at short wavelengths and from 830 to 850 nm at long wavelengths (and from 615 to 830 nm for the S‐cones). The principal reason for extending the range was to allow the cone fundamentals to be shifted along a log wavelength scale to model individual differences in cone *λ*_max_, but the extensions are  speculative and should be used with caution. The formulae also allow the effects of potentially large individual differences in macular, lens and photopigment optical densities on the cone fundamentals to be easily modeled. The resulting cone fundamental CMFs can then be straightforwardly linearly transformed to generate CMFs for real primaries whose power spectra are known or for imaginary primaries, such as **XYZ**, provided the relevant transformation matrix is known, and hence allow modeling of the effects of individual differences on those functions.

The uncertainties at wavelengths below 400 nm suggest the need for new measurements at longer UV wavelengths. However, any such measurements, and indeed any color matching predictions, in that region are complicated by the fluorescence of the lens and by the lens opacity especially in older observers (see above).

We have also provided a version of a common template shape to account for all three cone absorbance spectra. Given the assumption that the absorbance spectra should be shape invariant along a log wavelength scale, this can be usefully used to generate and investigate cone absorbance spectra and corneal spectral sensitivities for photopigments with different *λ*_max_. The common template should not be used to generate corneal cone spectral sensitivities or cone fundamentals if compliance with the CIE standard is required.

We have implemented the above equations in a computer program written in Python that can be used to generate cone fundamentals and CMFs for standard and individual observers. The program can be downloaded from http://github.com/CVRL-IoO/Individual-CMFs.git.

## AUTHOR CONTRIBUTIONS

Andrew Stockman did the original analysis and modelling, and prepared the first versions of the paper, figures and the Python code. Andrew T. Rider developed and improved the final versions of the paper and the Python code.

## Data Availability

Data openly available in a public repository that does not issue DOIs.

## Endnotes

* Note that substituting $l_{\mathrm{OD}}=0$ into Equation 6a implies ${\bar{l}}_{R}\left(\lambda \right)=0$, but we must consider the infinite dilution as a theoretical process. Mathematically, as a→0, $1-10^{-\mathrm{aX}}\to \mathrm{bX}$, where *b* is a scaling constant that depends on *a*. Accordingly, as $l_{\mathrm{OD}}\to 0$, ${\bar{l}}_{R}\left(\lambda \right)\to {\bar{l}}_{A}\left(\lambda \right)$ after normalization to unity peak that is, ${\bar{l}}_{A}\left(\lambda \right)$ should indeed be considered the spectrum of an infinitely diluted solution of the photopigment.

^†^
Note that although the CMFs are *defined* for test lights of equal energy, for practical reasons, such as the need to saturate the rods, the test lights in many experiments are not always of equal energy.

## APPENDIX A

**TABLE A1:** Summary of previous research into the changes in *λ*_max_ caused by amino acid substitutions in the L‐ or M‐cone opsin genes.

Exon	Codon	M opsin amino acids	L opsin amino acid s	Neitz and Neitz (2011)^a^ Review		Merbs and Nathans (1992)^b^ Recombinant pigments		Asenjo, Rim and Oprian (1994)^c^ Recombinant pigments		Merbs and Nathans (1993)^d^ Recombinant pigments	Sharpe et al. (1998)^e^ Psychophysics		Other data^f^ ERG, suction electrode	Suggested program Settings	
				M → L	L → M	M → L	L → M	M → L	L → M	L → M	M → L	L → M		M → L	L → M
2	116	Tyrosine	Serine	0	−2 to −3	0	−2.8 to −3.7	0	−4	–		−0.7		0	−3
3	180	Alanine	Serine	3	−3 to −4	3.4 to 4.2	−4.3	2	−4	−4.3 (4)		−2.4	L → M: −5	3	−4
4	230	Threonine	Isoleucine	3	−4	4	−2.6 to −2.7	4	−4	−1.2 (<1)		−2.7		3	−3
	233	Serine	Alanine					0	0	−0.4 (<1)				0	0
5	277	Phenyl‐alanine	Tyrosine	21	−21	15.1	−20.7 or −20.8	8	−8	−6.6 (7)		−26.4 or −26.1		7	−7
	285	Alanine	Threonine					15	−11	−13.7 (14)				14	−14
	309	Phenyl‐alanine	Tyrosine					0	0	−1.0 (<1)				0	0
Sum				27	−31	27.2	−26	29	−31					27	−31

^a^ Summary shifts from Figure 1 of a review article by Neitz and Neitz.^62^

^b^ Recombinant pigments and spectroscopy with shifts based on differences taken from Table 1 of Merbs and Nathans.^64^

^c^ Recombinant pigments and spectroscopy with shifts based on Figure 2 of Asenjo, Rim, and Oprian.^63^

^d^ Recombinant pigments and spectroscopy from Table 1 of Merbs and Nathans.^67^

^e^ Psychophysics determination of *λ*_max_ from table 5 of Sharpe et al.^7^

^f^ Neitz, Neitz, and Jacobs,^65^ Kraft, Neitz, and Neitz^68^ and Kraft (personal communication).
